# Radiomics-Based Classification of Clear Cell Renal Cell Carcinoma ISUP Grade: A Machine Learning Approach with SHAP-Enhanced Explainability

**DOI:** 10.3390/diagnostics15111337

**Published:** 2025-05-26

**Authors:** María Aymerich, Alejandra García-Baizán, Paolo Niccolò Franco, Mariña González, Pilar San Miguel Fraile, José Antonio Ortiz-Rey, Milagros Otero-García

**Affiliations:** 1 Diagnostic Imaging Research Group, Galicia Sur Health Research Institute, Hospital Álvaro Cunqueiro, 36312 Vigo, Spain; 2 Radiology Department, Hospital Álvaro Cunqueiro, 36312 Vigo, Spain; 3 Department of Diagnostic Radiology, IRCCS San Gerardo dei Tintori, Via Pergolesi 33, 20900 Monza, Italy; 4 Pathology Department, Hospital Universitario Álvaro Cunqueiro, 36312 Vigo, Spain

**Keywords:** radiomics, clear cell renal cell carcinoma, ISUP grade, machine learning, SHAP values, computed tomography, feature selection, texture analysis, non-invasive biomarkers

## Abstract

**Background**: Clear cell renal cell carcinoma (ccRCC) is the most common subtype of renal cancer, and its prognosis is closely linked to the International Society of Urological Pathology (ISUP) grade. While histopathological evaluation remains the gold standard for grading, non-invasive methods, such as radiomics, offer potential for automated classification. This study aims to develop a radiomics-based machine learning model for the ISUP grade classification of ccRCC using nephrographic-phase CT images, with an emphasis on model interpretability through SHAP (SHapley Additive exPlanations) values. **Objective**: To develop and interpret a radiomics-based machine learning model for classifying ISUP grade in clear cell renal cell carcinoma (ccRCC) using nephrographic-phase CT images. **Materials and Methods**: This retrospective study included 109 patients with histopathologically confirmed ccRCC. Radiomic features were extracted from the nephrographic-phase CT scans. Feature robustness was evaluated via intraclass correlation coefficient (ICC), followed by redundancy reduction using Pearson correlation and minimum Redundancy Maximum Relevance (mRMR). Logistic regression, support vector machine, and random forest classifiers were trained using 8-fold cross-validation. SHAP values were computed to assess feature contribution. **Results**: The logistic regression model achieved the highest classification performance, with an accuracy of 82% and an AUC of 0.86. SHAP analysis identified major axis length, busyness, and large area emphasis as the most influential features. These variables represented shape and texture information, critical for distinguishing between high and low ISUP grades. **Conclusions**: A radiomics-based logistic regression model using nephrographic-phase CT enables accurate, non-invasive classification of ccRCC according to ISUP grade. The use of SHAP values enhances model transparency, supporting clinical interpretability and potential adoption in precision oncology.

## 1. Introduction

Renal cell carcinoma (RCC) is one of the most common malignancies of the kidney, accounting for approximately 90% of all renal cancers. Within the RCC subtypes, clear cell renal cell carcinoma (ccRCC) is the most prevalent, constituting about 70–80% of RCC cases. The incidence of ccRCC has been increasing over recent decades and this trend underscores the need for improved diagnostic and prognostic tools for this disease [1].

Through medical imaging, renal lesions can be characterized using ultrasound (US), computed tomography (CT), or magnetic resonance imaging (MRI). However, CT and MRI are the most commonly utilized imaging techniques for the detailed characterization of renal lesions [2]. Using CT, the enhancement or contrast uptake of lesions is determined by comparing the Hounsfield units (HU) between the non-contrast phase and the post-contrast phase, with an increase of 20 HU between phases indicating lesion enhancement [3]. The nephrographic phase is optimal for the characterization and differentiation of renal lesions because it is during this phase that the enhancement of the renal parenchyma is maximal and most homogeneous, allowing for a more precise distinction between normal parenchyma and tumor lesions [4].

One of the critical challenges in managing ccRCC is the accurate determination of the International Society of Urological Pathology (ISUP) grade, which is a histopathological classification based on the degree of cellular differentiation and the presence of specific nuclear features. A variety of grading systems have been proposed to differentiate high-grade from low-grade ccRCC. that focuses on nuclear morphology. The Fuhrman grade was one of the most effective grading systems used in predicting the biological aggressiveness and metastatic potential of ccRCC. It is a 4-tiered grading system based primarily on the simultaneous assessment of nucleolar prominence, nuclear size, and nuclear irregularity. However, it was shown that this grading system was not very reproducible so a new grading system was proposed by the International Society of Urological Pathology (ISUP). The ISUP grading system is based on the evaluation of nucleoli features: grade 1 tumors have nucleoli that are inconspicuous and basophilic at ×400 magnification; grade 2 tumors have nucleoli that are clearly visible at ×400 magnification and eosinophilic; grade 3 tumors have clearly visible nucleoli at ×100 magnification; and grade 4 tumors have extreme pleomorphism or rhabdoid and/or sarcomatoid morphology [5,6,7].

This classification closely correlates with patient prognosis and is pivotal for therapeutic decisions. Thus, the possibility of non-invasively determining the ISUP grade through advanced imaging techniques could have a high impact on the management of ccRCC [8]. However, establishing the ISUP grade through medical imaging is still not feasible.

Radiomics has emerged as a promising tool in the classification of several pathologies through the quantitative analysis of features extracted from medical images [9]. This approach involves extracting a large number of quantifiable features from radiological images that can be used to create predictive models using machine learning techniques [10]. For example, radiomics has shown utility in assessing tumor phenotype in the lungs and brain [11,12] or in predicting treatment response in breast cancer [13], among others.

In the context of RCC, several studies have explored the use of radiomics to classify these tumors [14,15,16,17,18]. In particular, research has demonstrated that radiomic features derived from computed tomography (CT) can significantly discriminate between different ISUP grades in ccRCC [19,20,21,22]. These findings suggest that radiomics could serve as a non-invasive method for assessing the ISUP grade, providing critical information for treatment planning and prognostic evaluation. Moreover, radiomics holds potential in the prediction of survival of these patients [23] or in the prediction of mutations [9].

However, one of the main challenges associated with radiomic models is their explainability. The complexity of machine learning models often results in a “black box” where the model’s decisions are difficult to interpret. This lack of transparency can limit the clinical acceptance of these models and raise ethical and legal issues in medical decision-making [24]. Therefore, it is crucial to develop methods that enhance the interpretability of radiomic models to ensure their effective implementation in clinical practice. In this context, SHAP values (SHapley Additive exPlanations) are a powerful tool for enhancing the explainability of machine learning models, allowing the identification of which features have the most significant impact on the model’s predictions [25].

Previous research using multiphase CT radiomics has shown variable success in ccRCC grading, but most studies lack interpretability mechanisms. Our study focuses on single-phase nephrographic CT and incorporates SHAP values for model explainability, addressing this gap. The use of radiomics to classify ccRCC according to the ISUP grade is a promising area of research that could significantly improve the management of this disease. Accurate non-invasive prediction of ISUP grade in ccRCC is clinically crucial, as it directly influences prognosis and therapeutic strategies, yet remains challenging with current imaging modalities. However, addressing the challenges of explainability is essential to maximize the clinical impact of these advanced technologies. In this work, an ISUP grade classification model for ccRCC will be developed based on radiomic features extracted from the nephrographic phase of CT scans. Previous methods of feature selection will be applied, and once the best model is developed, the importance of each variable will be explained using SHAP values.

## 2. Material and Methods

### 2.1. Demographics

**Figure 1:**
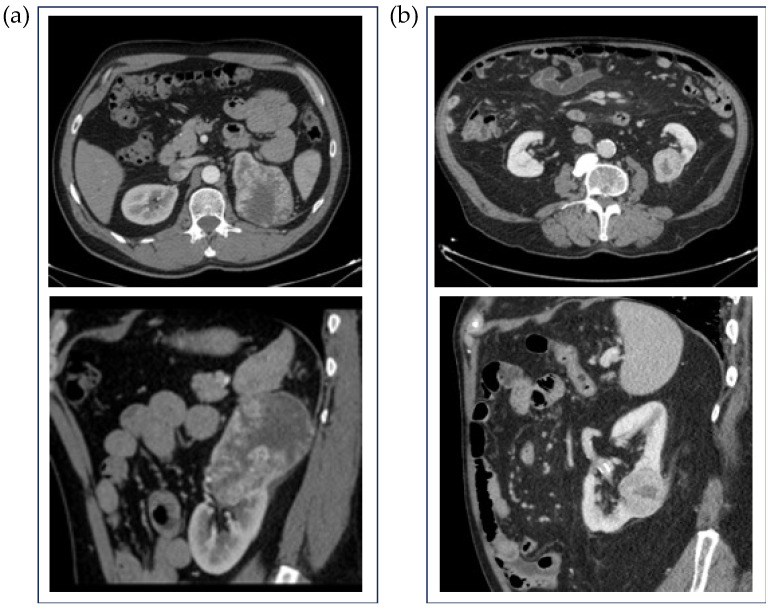
CT axial and sagittal sections of the nephrographic phase of two subjects show heterogeneously enhancing masses at the upper pole (**a**) and middle third (**b**) of the left kidney. After nephrectomy, the histopathologic results demonstrate that both lesions were ccRCC with a low (**a**) and high (**b**) ISUP grade. Imaging features do not allow the identification of different degrees of histopathological aggressiveness.

This retrospective single-center study included 173 patients with solid renal masses identified on CT imaging performed between 2016 and 2021. The study has been approved by the local ethics committee under the code 2021/008. All lesions have histopathological confirmation, which is considered the gold standard. Exclusion criteria included incomplete imaging studies or the absence of histopathological confirmation. Subjects included in the study had a single renal lesion that had not been previously treated. Out of the total database, 17 masses corresponded to papillary RCC type I, 9 to papillary type II, 19 to chromophobe RCC, and 19 to renal oncocytomas. From the initial cohort, 109 patients with histopathologically confirmed clear cell RCC were selected for radiomic analysis. Of these, 42 were classified as low ISUP grade (1 or 2) and 67 as high ISUP grade (3 or 4). Table 1 shows the demographic variables of the selected cases. Figure 1 displays an axial section of the nephrographic phase of a CT study of a low ISUP grade ccRCC and a high ISUP grade ccRCC.

### 2.2. Image Acquisition

The selected CT imaging study was the one where the solid renal mass was initially detected, often incidentally. The nephrographic phase (90 s post-contrast) of the initial diagnostic CT was selected for each case. CT scans were performed using various multi-detector scanners from Siemens, GE, and Philips, as part of routine clinical protocols.

### 2.3. Image Analysis

All images were anonymized and uploaded to Quibim Precision 2.8 (Quibim S.L., Valencia, Spain), a CE-marked and IBSI-compliant radiomics platform. The lesion volume of interest (VOI) was manually segmented on each nephrographic-phase image. Radiomic features from each VOI were extracted using commercial software. Intensity normalization, isotropic resampling to 1 × 1 × 1 mm^3^, and outlier removal were applied before feature extraction. Distance to neighbour was set to 1.

A total number of 106 radiomic features were calculated and they were grouped in seven families: shape, first order, gray-level co-occurrence matrix (GLCM), gray-level run-length matrix (GLRLM), gray-level size-zone matrix (GLSZM), gray-level dependence matrix (GLDM), and neighbouring gray-tone difference matrix (NGTDM). The complete list of features is shown in the Supplementary Materials.

### 2.4. Feature Selection

The intraclass correlation coefficient (ICC) was calculated for each radiomic feature based on manual segmentations performed independently by three radiologists (resident, junior, and senior levels). Only features with ICC ≥ 0.90 were retained. Each radiologist conducted a volumetric segmentation of the lesions, and the radiomic features were extracted using the Quibim platform. ICCs among the three radiologists were computed using the ICC function from the irr package, with the model set to “two-way” and the type set to “agreement”. Depending on the ICC values, the correlation was considered excellent (ICC ≥ 0.90), good (0.75 ≤ ICC < 0.90), moderate (0.50 ≤ ICC < 0.75), or poor (ICC < 0.50). Only excellent features were selected. Statistical analysis was conducted using R software (version 4.0.2). Highly correlated features (Pearson’s r ≥ 0.9) were excluded to reduce redundancy. The minimum Redundancy Maximum Relevance (mRMR) algorithm was then applied to select the most informative, non-redundant features. This algorithm selects features that have the maximum relevance with the target variable while ensuring minimum redundancy among the features. It was employed to further refine the feature set by identifying those variables that contribute the most to the predictive power of the model. Python software (3.11.2 version) was employed for these selections.

### 2.5. Model Elaboration and SHAP Values

Once the radiomic features were selected, the dataset was split into training and test groups in an 80–20% proportion. Data were normalized, and cross-validation parameter was set to 8. The model’s performance remained consistent across different cross-validation schemes, with similar f1 and accuracy values, choosing 8-fold as a reasonable compromise between fold size and validation robustness. Other validation strategies, such as Leave-One-Out Cross-Validation (LOOCV), were discarded due to their considerable variability in performance across iterations. The following machine learning models were trained: support vector machine (SVM), random forest (RF), and logistic regression (LR). Grid search space was defined to optimize the hyperparameters of the models and f1 metric was used as the score parameter. For each model appropriate parameter grids were explored, including variations in regularization strength, kernel types, tree depth, and solver methods. Once the best-performing hyperparameter combination was found, the models were applied to the test subset and the performance of each one was evaluated. In the classification report, metrics like accuracy; f1-score, precision, and recall, among other parameters, were presented. The receiver operating characteristic (ROC) curves of the subsets and the area under the curve (AUC) with the 95% confidence interval were calculated.

To further understand the importance of each feature in the decision-making process of the best-performing model, SHAP (SHapley Additive exPlanations) values were used. SHAP values provide a unified measure of feature importance, explaining the output of the machine learning model by assigning an importance value to each feature for a particular prediction. This method helps in interpreting the model by illustrating how each feature contributes to the prediction, identifying the most influential radiomic features. For the elaboration of the machine learning models and the calculation of SHAP values, Python (3.11.2 version) was employed.

## 3. Results

**Figure 2:**
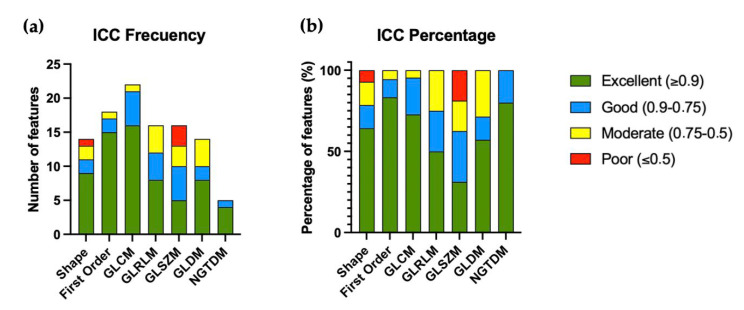
Distribution of Intra-Class Correlation Coefficient (ICC) values for different radiomic feature groups. Panel (**a**) shows the frequency of features within each group, while panel (**b**) displays the percentage of features in each category. Radiomic feature groups include Shape, First Order, GLCM, GLRLM, GLSZM, GLDM, and NGTDM.

In this section, the main results of the work are shown. No significant differences in demographic variables were observed between low and high ISUP grade ccRCC groups. First, the ICC was calculated for the 106 radiomic features from the segmentation performed by three radiologists with different levels of experience. The results are represented in Figure 2, and the data grouped by feature families are collected in Table 2.

Sixty-five radiomic features showed excellent reproducibility (ICC ≥ 0.90) across the segmentations by three radiologists. The highest ICC distributions were observed in the First Order and NGTDM families (83.3% and 80.0%, respectively) (Table 2).

After selecting the radiomic variables that meet an excellent ICC criterion among radiologists, the Pearson correlation matrix was calculated to eliminate highly correlated variables. The results are shown in Table 3.

**Figure 3:**
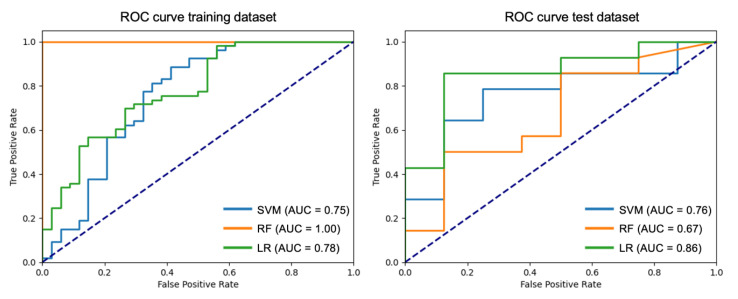
ROC curves for the different models on the training and test sets. The dotted line indicates the line of no discrimination in the classification.

As shown in Table 3, out of 65 radiomic features with excellent ICC, 20 features that were not highly correlated with each other were selected. Families of features, such as Shape or GLDM, had all their features correlated with each other, leaving one representative of each group. On the other hand, the features with excellent ICC from the NGTDM group were not correlated with each other and all of them are selected at this point. Next, using the mRMR algorithm, the four most relevant features for this work were selected. These were Major axis length (Shape), 10th percentile (First order), Large area emphasis (GLSZM), and Busyness (NGTDM). Collinearity was addressed through a sequential feature selection pipeline combining ICC filtering, Pearson correlation thresholding, and mRMR selection, which is considered sufficient to mitigate redundancy in this radiomic analysis. With these four features, three machine learning models were developed with their best hyperparameters and using 8-fold validation. The mean of the test scoring parameter f1 and standard deviation computed for each combination of hyperparameters across the eight folds were computed, obtaining 0.50 ± 0.06 for the support vector machine, 0.66 ± 0.09 for the random forest and 0.68 ± 0.06 for the logistic regression. To evaluate the models, a cross-validation using the best hyperparameter estimator was performed on the training set. The results are shown in Supplementary Materials. The metrics of the model’s performance on the test subset are shown in Table 4. Figure 3 shows the ROC curves for both the training and test subsets.

Logistic regression yielded the highest performance, with an accuracy of 82% and an AUC of 0.86 on the test set. The model’s AUC was 0.86 (95% CI: 0.71–1.00), outperforming SVM (AUC: 0.76) and random forest (AUC: 0.67). In order to provide greater explainability to the model and to establish the importance of the radiomic features in the prediction, the associated SHAP (SHapley Additive exPlanations) values were calculated. Considering that the selected model is inherently globally explained by its coefficients, SHAP-based explanations offer local interpretability by quantifying the contribution of each feature to individual predictions as well as confirming the global ranking of features derived from logistic regression coefficients.

**Figure 4:**
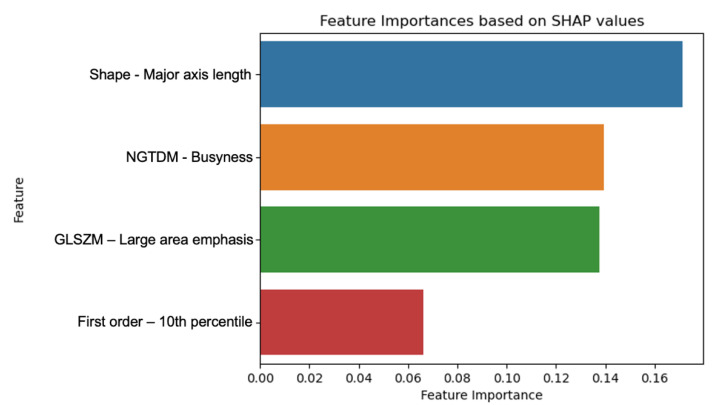
Histogram of the radiomic feature importance based on SHAP values for the classification of ccRCC according to high or low ISUP grade using logistic regression.

The graph shown in Figure 4 presents an analysis of the importance of each feature based on average SHAP values for the machine learning model that provides the best results in classifying ccRCC according to the ISUP grade. SHAP analysis identified major axis length (Shape) as the most impactful feature for ISUP grade classification, followed by busyness (NGTDM) and large area emphasis (GLSZM). These features were consistently associated with model predictions across both high- and low-grade examples, as illustrated by SHAP summary and local explanation plots.

**Figure 5:**
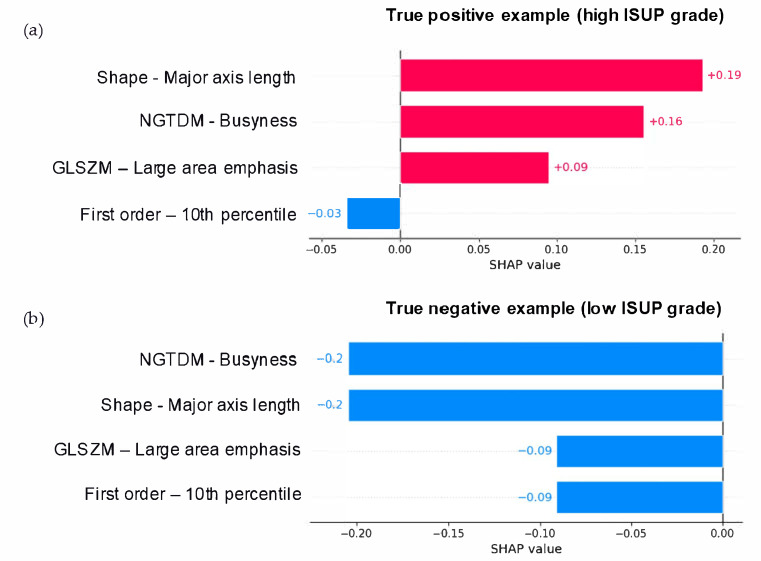
Local analysis of SHAP values for (**a**) a positive case (high ISUP grade) and (**b**) a negative case (low ISUP grade) correctly classified.

**Figure 6:**
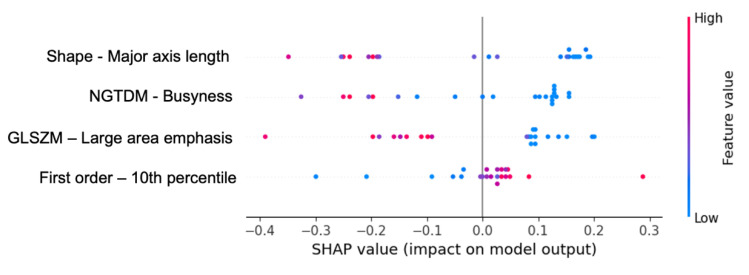
Beeswarm plot from the test dataset.

In Figure 5a, an example of a case correctly predicted as a high ISUP grade is shown. The features Major axis length, Busyness, and Large area emphasis, with different SHAP values, contributed to the high ISUP grade prediction (positive SHAP values). In Figure 5b, an example of a true low ISUP grade lesion, all the features have SHAP values that led the prediction towards a negative value, which in our classification corresponded to a low ISUP grade. Since SHAP values allow for the local analysis of all predicted cases, in the Supplementary Materials, examples of false positive and false negative cases are presented. Finally, Figure 6 presents the scatter plot showing the SHAP values for the most important features of the model.

In Figure 6, each point represents a SHAP value for each of the lesions analyzed in the test subset for a specific feature. The color code relates to the feature value and its position on the horizontal axis indicates its impact on the prediction towards a high ISUP grade (positive SHAP values) or a low ISUP grade (negative SHAP values). The dispersion of points along the horizontal axis shows the variability of the feature’s impact on the predictions. Generally, low values of the features Major axis length, Busyness, and Large area emphasis contributed to predicting high ISUP grades, while for the 10th percentile, high values did so.

The Major axis length (Shape) was the most influential feature in the model, suggesting that it was a strong predictor for distinguishing between high and low ISUP grade ccRCC. Regarding the high order or textural features, Busyness and large area emphasis also played a significant role, indicating that textural characteristics extracted from radiomic images were relevant for the accurate classification of ccRCC. Finally, first order features such as the 10th percentile, although less influential, also provided information that contributed to the robustness of the predictive model.

## 4. Discussion

This study demonstrates that a logistic regression model based on four radiomic features extracted from nephrographic-phase CT scans can accurately classify ccRCC by ISUP grade (accuracy: 82%; AUC: 0.86). The model was developed using a robust feature selection pipeline, including intraclass correlation coefficient (ICC) analysis, Pearson correlation filtering, and the mRMR algorithm. SHAP value analysis revealed that shape-related features, particularly major axis length, were the most influential, followed by textural descriptors. These results highlight the value of combining morphological and texture-based radiomic features to enhance both the performance and interpretability of machine learning models in ccRCC grading.

In this study, variable selection played a key role in optimizing model performance.

Among the selected features, major axis length emerged as the most predictive variable, reflecting the morphological heterogeneity of high-grade tumors. Busyness and large area emphasis, both textural features, also contributed substantially, suggesting that tumor disorganization and heterogeneity are radiomic correlates of aggressiveness.

Additionally, First Order features, such as the 10th percentile, reflect the intensity distribution within the image, providing valuable insights into tumor heterogeneity. Features from the NGTDM also played a significant role in classification. The Busyness feature, in particular, was one of the most determinant factors in predicting ISUP grade. This feature measures the complexity and variability in image texture, suggesting that higher-grade tumors exhibit more heterogeneous and disorganized textures compared to lower-grade ones. The combination of these feature families reinforces the usefulness of radiomics in the non-invasive assessment of ccRCC ISUP grade.

Yu et al. [26] performed the ccRCC ISUP grade classification using radiomic features extracted from multiple CT series to achieve better results than in the case of single-phase. The values they found by combining information from different series were similar to those of this work with a single phase. Additionally, their single-phase results showed that the nephrographic phase had the best classification metrics. On the other hand, in the study by Sun et al. [14], when working only with the nephrographic phase, the authors obtained AUC values of 0.56 compared to the 0.86 achieved in our work. Wang et al. [27] also used the combination of the corticomedullary and nephrographic phases in a multicenter study. Similarly to our work, logistic regression proved to be the best machine learning model for ISUP grade classification, although they reported low recall values of 0.58 compared to our work, which achieved 0.80. Xv et al. [28] performed the classification with a cohort of 406 subjects and using only the nephrographic phase, they achieved AUC values of 0.88. Nephrographic-phase CT-based radiomics may have a relevant clinical application given that it is the most frequently performed post-contrast phase in routine clinical practice. Kocak et al. [9] aimed to perform the classification from unenhanced CT images, but with AUC values not exceeding 0.71.

There is no general consensus on the CT protocol but the reference standard protocol for the study of renal masses is a triphasic study that is made up of a pre-contrast phase to determine the basal HU and assess possible contrast uptake (see if it also contains macroscopic fat); a corticomedullary phase at 40–70 s useful to differentiate RCC subtypes; a nephrographic phase 100–120 s where the maximum and most homogeneous enhancement of the parenchyma is observed, being able to identify smaller hypodense lesions and an excretory phase useful for planning surgery and being able to differentiate an RCC from urothelial carcinoma. In contrast to previous studies that rely on multiphase CT acquisitions or additional MRI sequences [29,30,31], our approach focuses exclusively on the nephrographic phase of CT imaging. By using a single-phase CT acquisition, our methodology increases the applicability of the predictive model to a broader patient population. In routine clinical practice, many patients do not undergo comprehensive multiphase CT protocols or combined CT and MRI evaluations, either due to institutional workflow limitations, patient comorbidities, or concerns regarding contrast media and radiation exposure. Therefore, models based on multiphase imaging inherently restrict their use to a subset of patients with complete imaging datasets, limiting their generalizability. In our case, we have only used the nephrographic phase because it can overlap with a portal phase, and many of the renal masses are small and found incidentally. Furthermore, according to the SAR (Society of Abdominal Radiology) guidelines, the recommended phases for the characterization of an indeterminate renal mass are the pre-contrast and nephrographic phases, leaving the corticomedullary and excretory phases as additional optional series. A key strength of our approach is the use of reproducible radiomic features (ICC > 0.98) combined with a minimal feature set, which enhances model robustness and interpretability. The exclusive use of the nephrographic phase reflects real-world imaging protocols and facilitates clinical integration.

Previous studies have used feature selection techniques similar to those employed in this study. ICC is a widely used selection criterion for various pathologies as it reduces the manual segmentation variability by filtering only those features that show excellent ICC in this pathology [14,32,33]. Interestingly, all features selected for the model development in this work had an ICC value above 0.98, which would indicate that the selected features are highly consistent and robust regardless of how the VOI of the lesion was labeled. The mRMR algorithm has also been used for feature selection in studies related to the use of radiomics for classifying the malignancy grade of ccRCC [34]. In several existing studies [16,26], feature selection for dimensionality reduction is also performed in a pre-filtering process. However, it would be interesting to explore options that consider incorporating feature selection within the machine learning model development itself in an embedded approach [35].

However, no studies were found in the literature that use SHAP values to explain the importance of features in the classification of ccRCC according to their ISUP grade. These values are an interesting tool that allows us to enhance the comprehensibility of the model by identifying which feature has the greatest impact on the predictions, aiding in the model’s interpretability. A certain degree of explainability in clinical decision support models is important for further acceptance and implementation of radiomics-based algorithms. Some areas where SHAP values have been used in radiomics models include radiation pneumonitis [36], spondylitis [37], and prostate cancer [38], among others. A key strength of our study is the integration of explainability through SHAP values, providing individualized insights into model predictions. This approach is scalable and independent of the specific machine learning algorithm used, allowing its application not only to logistic regression—which showed the best performance in our case—but also to any other predictive models that may demonstrate optimal performance in different settings. This ensures transparency and fosters greater clinical trust across various predictive frameworks. Local analysis using SHAP values is consistent in both correct and wrong predictions, so future research should consider including clinical variables or different biomarkers to reduce misclassification risk.

Since our model was developed in a population without prior therapies, its application in previously treated patients remains unexplored. This represents a relevant area for future research, as current evidence on the optimal management of recurrence remains limited [39]. Additionally, while our study did not assess metastatic progression, the prognostic relevance of the ISUP grade is well established. High-grade tumors are associated with increased metastatic potential and worse survival outcomes [40]. Recent radiomics studies have also demonstrated promising results for predicting metastatic behavior, both after surgical resection [41] and in patients with metastatic disease [42], supporting the potential future role of imaging biomarkers in personalized patient management.

This study has limitations, including its retrospective single-center design and limited sample size, which may restrict generalizability. The absence of external validation limits the robustness of the model across populations and scanners. Moreover, no harmonization techniques [43] were applied to reduce variability across the different CT scanners and should be considered to enhance generalizability. Future multicenter studies and multimodal imaging integration are needed to confirm these findings, following the guidelines established in the RQS [44], as well as the standardization practices recommended by IBSI [45] or EIBALL [46].

In conclusion, a radiomics-based logistic regression model using nephrographic-phase CT enables accurate, non-invasive ISUP grade classification in ccRCC. The use of SHAP values enhances model transparency and may facilitate clinical acceptance of AI-driven tools in renal oncology.

## Figures and Tables

**Table 1:** Demographic and clinical characteristics of clear cell renal cell carcinoma (ccRCC) cases stratified by ISUP grade. The table displays the distribution of sex, mean age, and laterality among patients with high and low ISUP grades.

		High ISUP Grade (n = 67)	Low ISUP Grade (n = 42)
Sex	Male (n = 84)	32 (85.1%)	52 (64.3%)
	Female (n = 25)	10 (14.9%)	15 (35.7%)
Mean age (years)		62.07 ± 11.91	63.35 ± 11.11
Laterality	Right (n = 59)	40 (59.7%)	19 (45.2%)
	Left (n = 50)	27 (20.3%)	23 (54.8%)

**Table 2:** Distribution of radiomic features across different ICC categories for the different feature groups. The groups with the highest number of features with excellent ICC are First order, with 83.3% and NGTDM, with 80.0%. GLCM, with 72.7% and Shape, with 64.3% also stand out. In contrast, the GLSZM group shows the poorest results, with 18.8% of the features with a poor ICC.

	Excellent (≥0.9)	Good (0.9–0.75)	Moderate (0.75–0.5)	Poor (≤0.5)
Shape (15)	9 (64.3%)	2 (14.3%)	2 (14.3%)	1 (7.1%)
First Order (18)	15 (83.3%)	2 (11.1%)	1 (5.6%)	0 (0%)
GLCM (22)	16 (72.7%)	5 (22.7%)	1 (4.6%)	0 (0%)
GLRLM (16)	8 (50.0%)	4 (25.0%)	4 (25.0%)	0 (0%)
GLSZM (16)	5 (31.3%)	5 (31.3)	3 (18.7%)	3 (18.7%)
GLDM (14)	8 (57.1%)	2 (14.3%)	4 (28.6%)	0 (0%)
NGTDM (5)	4 (80.0%)	1 (20.0%)	0 (0%)	0 (0%)

**Table 3:** Radiomic features that met an excellent ICC criterion and were not highly correlated with each other (Pearson threshold 0.9). In particular, 1 feature met the criterion from the Shape family, 5 from first order, 5 from GLCM, 2 from GLRLM, 2 from GLZSM, 1 from GLDM and 4 from NGTDM family.

Features After Applying Correlation (Threshold ≥ 0.9)	
Shape	Major axis length (ICC = 0.989)
First order	10 th percentile (ICC = 0.985) Entropy (ICC = 0.911) Maximum (ICC = 0.971) Minimum (ICC = 0.938) Uniformity (ICC = 0.921)
GLCM	Prominence (ICC = 0.964) Shade (ICC = 0.916) Contrast (ICC = 0.977) Inverse difference moment (ICC = 0.976) Inverse variance (ICC = 0.967)
GLRLM	Run length emphasis (ICC = 0.986) Gray-level non-uniformity (ICC = 0.990)
GLZSM	Large area emphasis (ICC = 0.983) Percentage (ICC = 0.963)
GLDM	Low dependence high gray level emphasis (ICC = 0.915)
NGTDM	Busyness (ICC = 0.984) Complexity (ICC = 0.961) Contrast (ICC = 0.945) Strength (ICC = 0.919)

**Table 4:** Metrics for the dataset used in testing the classification models trained with optimal hyperparameters.

Classification Metrics for the Test Subset					
Model	Accuracy	F1-Score	Precision	Recall	AUC (Confidence Interval)
Support Vector Machine (SVM)	0.73	0.69	0.71	0.71	0.76 (0.56–0.92)
Random Forest (RF)	0.64	0.61	0.61	0.61	0.67 (0.48–0.87)
Logistic Regression (LR)	0.82	0.80	0.80	0.80	0.86 (0.71–1.00)

## Data Availability

The datasets presented in this article are not available because of Local Ethics Committee restrictions. Nevertheless, the Phyton code and the requirements are openly available at https://github.com/mariaaymerichlopez/ccRCC_ISUP_grade/ (accessed on 12 April 2025).

## Supplementary Materials

The following supporting information can be downloaded at: https://www.mdpi.com/article/10.3390/diagnostics15111337/s1, Figure S1: SHAP-based explanations for two misclassified cases. (a) False negative: the model predicted ISUP low (class 0), but the true label was ISUP high (class 1). Most features strongly supported the incorrect class, highlighting a case with atypically low-grade radiomic appearance. (b) False positive: the model predicted ISUP high (class 1), but the true label was ISUP low (class 0). High values in shape and texture features led the model to overestimate the grade.; Table S1: Complete list of the radiomic features that were extracted with Quibim Precision software; Table S2: Cross-validation results using the best hyperparameters for each model (Support Vector Machine [SVM], Random Forest [RF], and Logistic Regression [LR]). The table reports the mean and standard deviation of the F1-score, accuracy, precision, and recall over 8 cross-validation folds on the training set, evaluating model robustness.
