# Supplementary material for: Radiomics-Based Classification of Clear Cell Renal Cell Carcinoma ISUP Grade: A Machine Learning Approach with SHAP-Enhanced Explainability
Source: Diagnostics (Basel). 2025 May 26;15(11):1337. doi: 10.3390/diagnostics15111337 (PMC12155185; doi:10.3390/diagnostics15111337)
Supplement: Supplementary file 1 [file diagnostics-15-01337-s001.zip › diagnostics-3601881-supplementary.pdf]

diagnostics

MDPI

Supplementary Material

List of radiomic features	
Shape	Elongation<br>Mayor axis length<br>Minor axis length<br>Sphericity<br>Flatness<br>Least axis length<br>Maximum 2D Diameter xy<br>Maximum 2D Diameter xz<br>Maximum 2D Diameter yz<br>Maximum 3D diameter<br>Volume<br>Area<br>Surface to volume ratio<br>Voxel volume
First Order	10th percentile
	90th percentile
	Energy
	Entropy
	Interquartile range
	Kurtosis
	Maximum
	Mean absolute deviation
	Mean
	Median
	Minimum
	Range
	Robust mean absolute deviation
	Root mean squared
	Skewness
	Total energy
	Uniformity
	Variance
Gray Level Cooccurrence Matrix (GLCM)	Autocorrelation
	Joint average
	Cluster prominence
	Cluster shade
	Cluster tendency
	Contrast
	Correlation
	Difference average
	Difference entropy
	Difference variance
	Joint energy
	Joint entropy
	Informational measure of correlation 1
	Informational measure of correlation 2

	Inverse difference moment
	Inverse difference moment normalized
	Inverse difference
	Inverse difference normalized
	Inverse variance
	Maximum probability
	Sum entropy
	Sum squares
Gray Level Run Length Matrix (GLRLM)	Gray level non uniformity
	Gray level non uniformity normalized
	Gray level variance
	High gray level run emphasis
	Long run emphasis
	Long run high gray level emphasis
	Long run low gray level emphasis
	Low gray level run emphasis
	Run entropy
	Run length nonuniformity
	Run length non uniformity normalized
	Run percentage
	Run variance
	Short run emphasis
	Short run high gray level emphasis
	Short run low gray level emphasis
Gray Level Size Zone Matrix (GLSZM)	Gray level non uniformity
	Gray level non uniformity normalized
	Gray level variance
	High gray level zone emphasis
	Large area emphasis
	Large area high gray level emphasis
	Large area low gray level emphasis
	Low gray level zone emphasis
	Size zone non uniformity
	Size zone non uniformity normalized
	Small area emphasis
	Small area high gray level emphasis
	Small area low gray level emphasis
	Zone entropy
	Zone percentage
	Zone variance
Gray Level Dependence Matrix (GLDM)	Dependence entropy
	Dependence non uniformity
	Dependence non uniformity normalized
	Dependence variance

	Gray level non uniformity
	Gray level variance
	High gray level emphasis
	Large dependence emphasis
	Large dependence high gray level emphasis
	Large dependence low gray level emphasis
	Low gray level emphasis
	Small dependence emphasis
	Small dependence high gray level emphasis
	Small dependence low gray level emphasis
Neighboring Gray Tone Difference Matrix (NGTDM)	Busyness
	Coarseness
	Complexity
	Contrast
	Strength

Table S1. Complete list of the radiomic features that were extracted with Quibim Precision software.

Model	SVM	RF	LR
F1	0.81 ± 0.05	0.80 ± 0.08	0.81 ± 0.06
Accuracy	0.74 ± 0.07	0.74 ± 0.10	0.74 ± 0.09
Precision	0.71 ± 0.07	0.74 ± 0.07	0.74 ± 0.08
Recall	0.98 ± 0.05	0.89 ± 0.14	0.91 ±0.09

Table S2. Cross-validation results using the best hyperparameters for each model (Support Vector Machine [SVM], Random Forest [RF], and Logistic Regression [LR]). The table reports the mean and standard deviation of the F1-score, accuracy, precision, and recall over 8 cross-validation folds on the training set, evaluating model robustness.

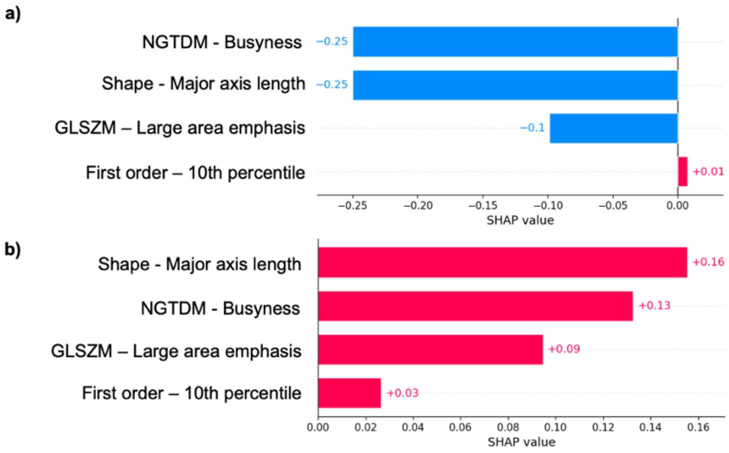

Figure S1. SHAP-based explanations for two misclassified cases. (a) False negative: the model predicted ISUP low (class 0), but the true label was ISUP high (class 1). Most features strongly supported the incorrect class, highlighting a case with atypically low-grade radiomic appearance. (b) False positive: the model predicted ISUP high (class 1), but the true label was ISUP low (class 0). High values in shape and texture features led the model to overestimate the grade.
